# COVID-19 restrictions on human rights in the light of the case-law of the European Court of Human Rights

**DOI:** 10.1007/s12027-020-00630-w

**Published:** 2020-10-06

**Authors:** Sanja Jovičić

**Affiliations:** Academy of European Law, Trier, Germany

**Keywords:** International Human Rights Law, ECHR

## Abstract

The aim of this article is to examine the restrictions imposed by European States on individual human rights during the COVID-19 pandemic in the light of the European Convention of Human Rights and Fundamental Freedoms. After an overview of the development of the case-law of the European Court of Human Rights on public emergencies and Article 15 of the Convention, the article will examine how the Court’s case-law could be applied to the current sanitary situation.

## Introduction

The current COVID-19 pandemic has strained the global economy and limited some of most important human rights and fundamental freedoms in democratic societies. Health safety restrictions have had an impact on freedom of liberty and security for persons being quarantined as a result of contracting or being suspected of having contracted the virus. Limits on freedom of expression have been imposed in order, allegedly, to prevent information disorder. Assemblies and protests have been prohibited to prevent the spread of the virus. Equally, access to courts has been impeded or allowed only under special arrangements. One might argue that there have been violations of the right to life of individuals who have died because of the virus and of the lack of sufficient medical care, especially in detention or care institutions. The right to family life has been disrupted due to restrictions on movement of persons across Europe. Furthermore, there have been instances of interference with the right to respect for private life by public authorities tracking infected persons. The emergency situation has caused an unprecedented chain of events affecting everyone and forcing States to take decisions restricting human rights within short time limits.

The aim of this article is to examine the restrictions imposed by European States during the COVID-19 pandemic in the light of the European Convention of Human Rights and Fundamental Freedoms. I will present the development of the case-law of the European Court of Human Rights on public emergencies and on Article 15 of the Convention and how it is currently applied by the Court. At the same time, I will examine the current COVID-19 restrictions put in place by a number of States Parties to the Convention, providing critical analysis and concrete recommendations for the future.

## Limitations and derogations in time of emergency under the European Court of Human Rights case-law

The Court examined cases relating to public emergencies either where a derogation of Art. 15 of the Convention applied or in the absence of such a derogation under the limitation clauses contained in some articles of the Convention, such as Arts. 8-11. In the absence of a limitation clause, the Court insisted on preserving some minimum requirements of a given right, for example under Arts. 5 and 6 of the Convention.^1^ It should be noted that when a Convention right contains a limitation clause there will be no need for a State Party to derogate from them as wider limitations may be imposed in times of public emergency already under the text of those provisions.^2^ States’ derogations up to date have concerned primarily Art. 5 of the Convention.

The Convention allows State Parties to derogate from human rights and fundamental freedoms protected therein under strict conditions set out in Art. 15 of the Convention. The plain text of this provision contains the following five elements:

1. **timing**: a derogation is possible only in time war or other public emergency threatening the life of the nation of a High Contracting Party;
2. **scope**: to the extent strictly required by the exigencies of the situation;
3. **respect for other international law obligations**: such measures must not be inconsistent with a State’s other obligations under international law (Art. 15(1));
4. **non-derogable rights**: certain human rights may not be derogated from (Art. 15(2)), namely the right to life under Art. 2 (except in respect of deaths resulting from lawful acts of war), the prohibition of torture, inhuman and degrading treatment and punishment under Art. 3, the prohibition of slavery and servitude under Art. 4(1) and the principle of *nulla poena sine lege* under Art. 7;
5. **obligation to inform**: State Parties are required to keep the Council of Europe fully informed of the measures which they have taken and the reasons therefore, as well as when such measures have ceased to operate and the Convention provision are again fully executed (Art. 15(3)).

Between March and April 2020, ten States Parties to the Convention notified the Secretary General of the Council of Europe of a derogation specifically with respect to the COVID-19 pandemic: Latvia, Romania, Armenia, the Republic of Moldova, Estonia, Georgia, Albania, North Macedonia, Serbia and San Marino.^3^ Albania,^4^ Latvia^5^ and North Macedonia^6^ each exercised a derogation in respect of Arts. 8 and 11 of the Convention, Arts. 1 and 2 of Protocol No. 1 and Art. 2 of Protocol No. 4. Estonia exercised a derogation from Arts. 5, 6, 8 and 11 of the Convention, Arts. 1 and 2 of Protocol No. 1, and Art. 2 of Protocol No. 4.^7^ Georgia derogated from Arts. 5, 8 and 11 of the Convention, Arts. 1 and 2 of Protocol No. 1, and Art. 2 of Protocol No. 4.^8^ Moldova derogated, in particular, from Art. 11 of the Convention, Art. 2 of Protocol No.1 and Art. 2 of the Protocol No. 4.^9^ Armenia appears to be limiting freedom of movement, the right to property, freedom of assembly and freedom of the press, whereby the media is obliged to report only official information.^10^ Romania exercised a derogation in respect of, *inter alia*, freedom of movement, the right to private and family life, the right to education, freedom of assembly, and the right to property.^11^ Additional measures were adopted to block “fake news” regarding the progress of COVID-19 in the mass media and online.^12^ Some of the above-mentioned countries have already withdrawn their notifications of derogation, including Albania, Estonia, Latvia, North Macedonia, Moldova, Romania and San Marino. Other member States adopted COVID-19-related emergency legislation without making a derogation under Art. 15.

Different cases concerning human rights violations in times of the pandemic might arise in the future before the Court. It is especially worrisome that many countries have put in place limits on freedom of expression. The question arises of whether those restrictions were necessary in the situation at hand. How much scope will State Parties now have to limit human rights in the light of the health crisis? Is the emergency legislation that has been adopted in line with the Convention? Were individual measures that were adopted a proportionate response to the situation? I will try answer these questions by examining the case-law of the Court applicable in public emergencies with and without a derogation under Art. 15 of the Convention. With regard to derogations, I will focus only on paragraph 1 of Art. 15.

### The existence of a public emergency

It is clear from the *travaux préparatoires* to the Convention in respect of Art. 15(1) that the drafters did not want restrictions imposed to be capable of being based on “reasons of State” and used to destroy democracy and the fundamental freedoms protected.^13^ In order to stress the exceptional nature of the circumstances justifying the application of Art. 15(1), the initial draft containing the wording “in time of war and other public emergency threatening the *interests of the people*”^14^ was subsequently replaced by the wording “in time of war and other pubic emergency threatening the *life of the nation*”^15^. This indicates that State Parties need to be cautious declaring a state of emergency only in exceptional circumstances, namely in times of war or when the very life of their nation is threatened.

Interestingly, the Court had opportunity to interpret Art. 15 of the Convention in its very first decision on the merits in *Lawless v. Ireland* (1961). In the context of IRA terrorist activities in Northern Ireland, the applicant was detained without being brought before a judge. The Irish Government notified the Council of Europe of a derogation under Art. 15. The Court clarified the wording “other public emergency threatening the life of the nation” as meaning “an exceptional situation of crisis or emergency which affects the whole population and constitutes a threat to the organised life of the community of which the State is composed”.^16^ The Court concluded that a “combination of several factors”^17^ pointed to the existence of a “public emergency threatening the life of the nation”, namely the existence of a violent secret army, operating outside the State’s territory and quickly, significantly increasing its terrorist activities.

Soon after in *the Greek case* (1968), the then filtering organ of the Court, the European Commission of Human Rights^18^, deciding upon a derogation of the Greek Government in the view of a *coup d’état* on its territory, developed the following four criteria to be considered when assessing the existence of a public emergency: 1) it should be actual or imminent; 2) the whole nation must be affected by it; 3) the continuance of the organised life of the community must be threatened; and 4) the crisis or danger should be “exceptional, in that the normal measures or restrictions, permitted by the Convention for the maintenance of public safety, health and order, were plainly inadequate.”^19^

In *Ireland v. the United Kingdom* (1969) the United Kingdom (UK) authorities detained without trial suspected terrorists who were ill-treated during their detention. The Court noted that Contracting States have a wide margin of appreciation to determine whether the life of their nation is threatened by a public emergency and the nature and a scope of derogations necessary to overcome the emergency by reason of “direct and continuous contact with the pressing needs of the moment”.^20^ However, such power is not unlimited and is subject to the supervision of the Court which will have a final say as to whether the measures taken State were “strictly required by the exigencies of the situation”.^21^ It is important to note that the Irish Government argued that it had been *ex post facto* clear that the extrajudicial detention carried out by the UK authorities had not been absolutely necessary. The Court replied that it “must arrive at its decision in the light, not of a purely retrospective examination of the efficacy of those measures, but of the conditions and circumstances reigning when they were originally taken and subsequently applied” 22. Whether those measures were effective will, therefore, not play a major role in the Court’s assessment, but, in particular, whether the circumstances at issue called for the adoption of such measures. One must not take the stand of a future observer but of one at the time of the facts of the case when immediate action was required by the State under unforeseen and/or uncontrollable circumstances. It should further be noted that although the initial problem concerned prolonged detentions without access to a court, the respondent Government was allowed under Art. 15 to effect progressive adaptations of the emergency measures such as the release of the detainees, gradual access to judicial or semi-judicial remedies.^23^

Forty years later the UK made another derogation under Art. 15 in order to detain without charges foreign nationals suspected of al-Qaeda-related terrorist activities pending their deportation. The Court examined the derogation in *A. and Others v. the United Kingdom* (2009). In line with the “wide margin of appreciation” doctrine, the Court gave special weight to the findings of the domestic courts as to the existence of the emergency.^24^ It interpreted the notion of “imminence” widely. A State is not required “to wait for disaster to strike before taking measures to deal with it”.^25^ The measures in question do not have to be “temporary”, although “the proportionality of the response may be linked to the duration of the emergency”.^26^ Indeed, the Court has previously dealt with public emergencies which lasted for years, as in the case of Northern Ireland. The Court further took into account a much broader range or factors, whereby the work of State institutions does not have to be endangered.^27^ The fact that other member States did not make use of Art. 15 of the Convention in the fight against terrorism was not of importance to the Court as each State (and more especially its national courts) is best placed to assess the evidence as to the existence of a public emergency.^28^

The cases involving a public emergency dealt by the Court have so far concerned either terrorist activities or armed conflicts. Although the Court has not dealt with a health emergency such as what we are facing at the moment, its case-law is easily applicable to the current COVID-19 situation. Being a highly infectious disease potentially leading to death^29^, COVID-19 has required the adoption of special measures to contain the virus, such as restrictions on the freedom of movement, liberty and security, the right to work and the right to education. These restrictions have affected the very functioning of societies and have threatened the life of whole nations, as already acknowledged by the organs of the Council of Europe.^30^ Medical professionals continue to warn that the situation is not expected to be temporary and are unable to predict when exactly it will end. Although some restrictions on individual rights adopted at the beginning of the crisis have slowly been eased, Governments are ready to re-implement them in the event of a rise in the number of infections. Taking into account the wide margin of appreciation, the Court will attach particular importance to national courts’ findings in its eventual future assessment of the public emergency at issue in the light of a wide range of elements, be these the high number of persons requiring hospitalisation, the failure of a domestic health system, the need to prevent the further spread of the virus from the most affected countries or any other actual exceptional circumstance affecting the life of a nation.

### Strictly required by the exigencies of the situation

In *Lawless* the Court examined whether general and individual measures of detention without trial were “strictly required by the exigencies of the situation”. In this case, peace and order could not be restored by means of ordinary law and by the domestic courts. The Court considered the nature of IRA activities, restrictions on cross-border gathering of evidence and the possible repercussions of a complete closure of borders on the population.^31^ The Court found that several safeguards had been put in place to prevent abuse, namely the constant supervision of Parliament, the establishment of a Detention Commission which consisted of two judges (and an officer of the armed forces) which was able to order the release of persons from detention with binding effect upon the Government. Furthermore, the Irish Government publicly announced that those who undertook to comply with national law and refrain from prohibited activities would immediately be released.^32^

In *Brannigan and McBride v. The United Kingdom* (1993), the Government exercised a derogation under Art. 15 in order to keep the extended period of detention of suspected terrorists and avoid a violation of Art. 5. The applicants were detained soon after the derogation and their detention lasted approximately four and six days, respectively, based on an order of the executive and on secret information not disclosed to the applicants. No judicial authority was involved in the decisions on detention (Art. 5(3)).^33^ The Court noted that it “must give appropriate weight to such relevant factors as the nature of the rights affected by the derogation, the circumstances leading to, and the duration of, the emergency situation”.^34^ The Court accepted that, notwithstanding a decreasing level of violence over the years, an emergency situation still persisted in Northern Ireland. On the issue of whether the measures were “strictly required by the exigencies of the situation”, the Court accepted that the derogation had been genuine in the view of special difficulties associated with the investigation and prosecution of terrorist crime. As to the absence of judicial control over the extended detention, the Court noted that Art. 5(3) does not necessarily require to have a judge or judicial authority involved in the decisions on (extension of) detention, but a “procedure that has a judicial character although that procedure need not necessarily be identical in each of the cases where the intervention of a judge is required”.^35^ The Court accepted the Government’s argument that in the light of the special circumstances prevailing in Northern Ireland at the time, where the judiciary was small and vulnerable to terrorist attacks, allowing the courts to decide on information which was not be disclosed to the detainee would have undermined the public confidence in the independence of the judiciary.^36^ When examining whether safeguards against abuse had been put in place, the Court noted that the applicants could avail themselves of the remedy of *habeas corpus* to review the lawfulness of their arrest and detention, that they could access a lawyer after 48 hours from the time of arrest and that such access could be denied on reasonable grounds subject to judicial review. Moreover, there had been a right to inform a relative or friend of the detention, access to a doctor had always been granted, and the legislation at issue was subject to independent and regular review.^37^

During the same period Turkey exercised a derogation under Art. 15 in respect of Art. 5, following the establishment of emergency rule in the provinces where clashes between its armed forces and the members of the Workers’ Party of Kurdistan (PKK) were taking place. The derogation was examined in *Aksoy v Turkey* (1996), which concerned the applicant’s having been taken into custody and his ill-treatment by the police on suspicion of terrorist activity. The applicant’s detention lasted fourteen or more days without him being promptly brought before a judge or a judicial authority. The Turkish Government tried to justify the applicant’s prolonged detention with the difficulties in investigating terrorist offences which covered a wide area in the country. No particular argument was adduced as to why the domestic courts had been unable to review the lawfulness of suspected terrorists’ detention.^38^ For the Court, the period of detention of fourteen days was “exceptionally long” and the Government had not provided detailed reasons justifying the complete absence of judicial supervision,^39^ as had been done by the UK Government in *Brannigan and McBride*. Furthermore, no sufficient safeguards against abuse had been put in place as the applicant had had no access to a lawyer, doctor, relative or friend. This was coupled with the absence of any possibility of testing the lawfulness of his detention - an absence which had allowed the ill-treatment of the applicant.^40^

In the abovementioned *A. and Others v. the United Kingdom* case the applicants were foreign nationals against whom a deportation order had been issued on security grounds, but who could not be deported as they would have run the risk of ill-treatment. During the period of detention, no action was taken with a view to deporting them. The applicants could challenge their deprivation of liberty before a special appeals commission which would base itself both on open and closed material which, for reasons of national security, would not be disclosed to the applicants and their lawyers. The closed material could be challenged before the Commission by a special advocate acting on the behalf of the applicants. This counsel could discuss with the applicants only the open statements and evidence. The highest UK court found that there had existed an emergency situation, yet the measures taken by the Government had not been strictly required by the exigencies of the situation. There had been an element of bad faith from the side of the Government, in that the contested immigration measures had actually been security measures discriminating between nationals and non-nationals which led the Court to conclude that there had been a violation of Art. 5(1)(f) of the Convention.^41^ The applicants further complained that the procedure to challenge the lawfulness of their detention had not been fair, contrary to Art. 5(4) of the Convention. Although the UK did not derogate from this provision, the Court still took account of the terrorist threat posed by al-Qaeda and its associates the UK.^42^ However, even in those circumstances, the Court held that Art. 5 § 4 entails “substantially the same fair-trial guarantees as Art. 6 § 1 in its criminal aspect”^43^ and that the applicants had to have a possibility of effectively challenging their detention^44^. For the Court it was important to determine whether the “open material played the predominant role in the determination” and whether the “allegations contained in the open material were sufficiently specific” to enable the applicant to challenge them effectively.^45^

Most recently, in *Mehmet Hasan Altan v. Turkey* (2018), after the attempted military coup in Turkey in 2016, a journalist was put in pre-trial detention on suspicion of having supported the terrorist organisation deemed responsible for the coup. Prior to the applicant’s detention, the Turkish Government made a valid derogation under Art. 15 of the Convention.^46^ Although the Turkish Constitutional Court found that the applicant’s pre-trial detention had had no factual basis, the lower court refused to release the applicant. In finding the applicant’s detention to have been unlawful under Art. 5(1) of the Convention, the Court endorsed the reasoning of the Constitutional Court that even in times of emergency detention had to be justified with sufficient evidence.^47^ It was especially striking for the Court that, contrary to the principle of rule law, a binding decision of the domestic highest judicial authority had not been respected, which added to the arbitrariness of the applicant’s detention.^48^ The applicant further complained that he had had no access to the case file to challenge his pre-trial detention. The Court accepted the Government’s contention that the applicant, who was assisted by a lawyer, had acquired sufficient knowledge of the substance of the evidence through the detailed questions of the prosecuting and judicial authorities which were also reproduced in the relevant records.^49^ On a further complaint about the lack of speediness of the proceedings before the Constitutional Court (which had taken fourteen months and three days), the Court accepted that the significant increase in the Constitutional Court’s backlog of cases as a result of the public emergency and the complexity of the case at issue justified such a long decision-making process.^50^ However, the Court immediately emphasised that this was an exceptional situation and that it would continue to have a final say as to the “speediness” of the detention.^51^ The applicant complained lastly that the pre-trial detention violated his freedom of expression under Art. 10 of the Convention. The Court relied heavily on the reasoning of the Constitutional Court which had found that the detention at issue had had no factual basis and had had a chilling effect on freedom of expression and the press.^52^ Evidence was presented to the effect that in the case of journalists the Turkish judiciary had interpreted the emergency legislation too widely.^53^ The Court stressed that States were entitled to take measures against incitement to violence, especially in the aftermath of a military coup which threated the core values of a democratic society.^54^ However, the public emergency “must not serve as a pretext for limiting freedom of political debate”, and “any measures taken should seek to protect the democratic order from the threats to it, and every effort must be made to safeguard the values of a democratic society, such as pluralism, tolerance and broadmindedness”.^55^ “Criticism of governments and publication of information regarded by a country’s leaders as endangering national interests should not attract criminal charges for particularly serious offences such as belonging to or assisting a terrorist organisation, attempting to overthrow the government or the constitutional order or disseminating terrorist propaganda”.^56^ Moreover, “pre-trial detention should only be used as an exceptional measure of last resort when all other measures have proved incapable of fully guaranteeing the proper conduct of proceedings. Should this not be the case, the national courts’ interpretation could not be regarded as acceptable.”^57^ It should be noted that in normal circumstances, the Court does not appreciate criminal sanctions being applied in respect of exercises of freedom of expression as such sanctions inevitably have a chilling effect.^58^ In the past, the Court has been prepared to accept such sanctions only in the context of the remarks supporting a terrorist organisation.^59^ However, in *Mehmet Hasan Altan v. Turkey* the Court emphasised with very strong language that notwithstanding an emergency situation the member States would have to provide a very good justification for detaining and prosecuting someone in the exercise of his or her freedom of expression.

Within the same context in *Alparslan Altan v. Turkey* (2019) the Court was called to rule on the lawfulness of the detention of a Constitutional Court judge. The Court concluded that the new extensive interpretation of the relevant domestic criminal provisions allowing the detention of judges while disregarding the special procedure applicable to them, resulted in the applicant’s detention not being “in accordance with a procedure prescribed by law” under Art. 5(1) of the Convention.^60^ The Court paid special attention to the fact the applicant was a judge who was to be afforded special protection, justified by the need to safeguard the independence of the judiciary.^61^ In the public emergency at issue, the Court required the “lawfulness” criterion of Art. 5 to be respected in the same way as in normal situations, where the necessary clarity and foreseeability of the law must be preserved. The way the Constitutional Court applied the domestic law in the case at issue was not in compliance with the principle of legal certainly and was further “manifestly unreasonable”.^62^ The Court further pointed out that, even in the special circumstances of the case right after the attempted coup, evidence showing the existence of a reasonable suspicion of having committed the alleged offence must be present at the time when detention is ordered.^63^ Despite evidence showing the applicant’s alleged involvement in the terrorist organisation having been presented after his initial detention, the Government was not dispensed from showing the “reasonable” of the suspicion at the time of the decision on detention, which “forms an essential part of the safeguard laid down in Article 5 § 1 (c)”.^64^

Similarly, in *Baş v. Turkey* (2020), a judge had been detained and convicted for alleged membership of the terrorist organisation responsible for the military coup in Turkey. In assessing the alleged lack of reasonable suspicion that the applicant had committed an offence, although noting the serious security situation where even some members of the judiciary appeared to been involved in the endeavours to overthrow the Government, the Court refused to overstretch the notion of “reasonableness” on the grounds that this would result in the destruction of the very essence of the safeguard secured by Art. 5(1)(c).^65^ The Court held that apart from general information, no concrete evidence directly and personally related to the applicant had been taken into account by the domestic courts when ordering his detention.^66^ The applicant further complained that the fact that he had not appeared before a court for one year and two months after the decision on his detention had been in breach of Art. 5(4) of the Convention. The Court noted that the attempted military coup was a “contextual factor which it must fully take into account in interpreting and applying Art. 15 of the Convention in the present case”.^67^ In the Court’s view, in the first few months after the coup attempt a derogation from Art. 5 might have been justified. However, with the passage of time the public emergency considerations became less relevant and of a lower intensity, thus making the Court’s assessment of the exigencies of the situation stricter.^68^ The Court clearly indicated an obligation on State Parties to justify derogations from Art. 5 during the whole period of the detention.

Lastly, in *Kavala v. Turkey* (2019) the Court examined the detention of a human rights defender who was suspected of attempting to overthrow the Government and constitutional order, as the instigator and leader of the Gezi Park events 69 and as a participant in the 2016 attempted coup. On 18 July 2018, when the applicant was still detained, Turkey lifted the state of emergency.^70^ When assessing the reasonableness of the suspicion that the applicant had committed the acts alleged by the prosecution, the Court found no evidence showing that those acts involved violence or force, but represented non-violent acts performed in the exercise of the Convention rights.^71^ “The very fact that such acts were included in the bill of indictment as the constituent elements of an offence in itself diminishes the reasonableness of the suspicions in question.” 72 This was one of the factors which led the Court to conclude that the Government acted in bad faith under Art. 18 of the Convention and that the applicant’s detention was part of campaign pursued by the Government to silence human rights defenders.^73^

In the light of the importance of Art. 5 of the Convention to protecting individuals against arbitrary detention, the Court has never accepted extensive derogations from this right. On the contrary, it has insisted on preserving minimum requirements and safeguards against abuse. While allowing longer periods of detention in emergency situations, the Court rejected restrictions on judicial control over detention. The Court further reinforced the protection afforded by Art. 5 by linking its procedural requirements in para. 4 to Art. 6(1), by requiring reasonable suspicion to be based on facts present when the detention was ordered, by maintaining the requirement that the detention be lawful and, most importantly, by requiring that individuals genuinely be detained for the purposes indicated by the Government. Art. 10 of the Convention seems to be applicable in full even when being derogated from given its fundamental importance for democracy. Evidently, the Court’s interpretation of Art. 15 is becoming ever stricter. Only in two cases (which are more than thirty years old), has (one) State successfully invoked this Article. Domestic authorities will have to take very seriously the criterion of “strictly required by the exigences of the situation”, and take into consideration that the Court will carefully examine the reasoning provided by a State Party as to why the ordinary law is not capable of effectively dealing with the emergency situation,^74^ and take into consideration the nature of the right interfered with, as well as the circumstances and the duration of the emergency.

### The assessment of public emergencies without a derogation

In *Brogan and Others v. the United Kingdom* (1988), the Court examined the case of suspected terrorists who had been arrested without charge and had not been brought promptly before a judicial authority within seven days as required under Art. 5(3) of the Convention. The UK Government had already withdrawn its notice of derogation under Art. 15 when the applicants’ arrest took place. Nevertheless, the Court took into account the still extant problem of terrorism in the respondent State.^75^ However, those special circumstances could not justify the impairment of “the very essence of the right guaranteed by Article 5 para. 3”,^76^ which includes the obligation to release or bring a person promptly before a judicial authority. The Court refused to give an extensive interpretation to the word “promptness” in Art. 5(3) and concluded that even the shortest period of detention of four days and six hours without judicial control violated that provision.^77^ What is important to note in this case is that the Court, in the absence of a derogation under Art. 15, and while considering the special circumstances of an emergency situation, held that the minimum requirement of “being promptly brought before a judicial authority” under Art. 5 must be secured.

In the following years, as mentioned above^78^, a series of cases against Turkey have arisen in connection with PKK terrorist activity. These cases called for the examination of several human rights against the background of a sensitive security situation. Art. 5 complaints were examined in *Sakik and Others v. Turkey* (1997), where the applicants were subject to prolonged detention without judicial supervision and were sentenced for alleged terrorist activities pursuant to emergency legislation. Refusing to apply a derogation,^79^ the Court held that the investigating authorities did not “have *carte blanche* under Article 5 to arrest suspects for questioning, free from effective control by the domestic courts and, ultimately, by the Convention supervisory institutions, whenever they choose to assert that terrorism is involved.” 80 In the Court’s view the applicants’ detention for periods of between twelve and fourteen days without judicial supervision was too long and in breach of Art. 5.^81^

With respect to Art. 10 complaints during emergency situations, the Court stressed in *Zana v. Turkey* (1997) that the general principles of Art. 10 applied in full to situations where national security and public safety were endangered.^82^ The Court considered that the statements of the mayor of a region faced with terrorism to a large newspaper could have exacerbated violence and that the domestic courts provided sufficient and relevant reasons for his criminal conviction.^83^ Likewise, *Sürek v. Turkey* (1999) concerned the criminal conviction of the owner of a newspaper for dissemination of separatist propaganda. The Court recalled “that there is little scope under Article 10 § 2 of the Convention for restrictions on political speech or on debate on matters of public interest”.^84^ However, where such remarks incite violence, State Parties enjoy a “wider margin of appreciation when examining the need for an interference with freedom of expression”.^85^ Taking into account, *inter alia*, the words used by applicant amounting to hate speech and the glorification of violence, his position and the relatively low fine imposed on him, the Court held that the interference under Art. 10 was proportionate to the legitimate aim pursued.^86^

An Art. 2 complaint was the subject-matter of *Al-Skeini and Others v. the United Kingdom* (2011). In this case the Court was called to decide upon the killing, ill-treatment and disappearance of Iraqi nationals during security operations carried out by the armed forces of the UK as an occupying power in Iraq. The Court accepted that in the aftermath of the military invasion of Iraq, the civilian infrastructure, including the law enforcement and criminal justice systems, was not functioning.^87^ Nonetheless, the Court held that “even in difficult security conditions, all reasonable steps must be taken to ensure that an effective, independent investigation is conducted into alleged breaches of the right to life.”^88^ A similar conclusion was reached in the case of *Jaloud v. The Netherlands* (2014) where the Court found serious deficiencies in the investigation of the shooting of an Iraqi national by Netherlands servicemen at a vehicle checkpoint.^89^

A complaint under Art. 6 was recently raised in *Khlebik v. Ukraine* (2017) where the applicant had been prevented from having his appeal against his criminal conviction examined before a higher court as the domestic courts had no access to his case file in the territory of Luhansk where Ukraine had lost control due to ongoing armed violence. Ukraine had notified the Council of Europe of a derogation from Art. 6 of the Convention applicable in the territories where it had lost control. However, according to both parties this derogation was not applicable to the applicant’s case as it had been adopted after the facts of his case occurred. The validity of the derogation was, therefore, not examined by the Court.^90^ Nevertheless, the Court decided to analyse the case in the light of the general situation in Ukraine, especially the ongoing hostilities in the non-Government controlled territories where the applicant’s case-file was located.^91^ In the view of “objective obstacles” preventing the Government from securing the file and, in particular, the applicant’s release based on a more generous interpretation of domestic law, the Court concluded that no violation of Art. 6 had occurred.^92^

In the absence of a (valid) derogation under Art. 15, the Court has been prepared to take into account the exceptional circumstances of a public emergency. On the one hand, the Court requires that the very essence of Art. 5 be preserved, including the requirement of lawfulness and the absence of arbitrariness of the detention. On the other hand, there may be exceptions to Arts. 6 and 10 in the light of all of the special circumstances of a case. It should, however, be noted that the above-mentioned cases were context-specific as all related to armed violence and, in some cases, to extraterritorial acts of State. It might, therefore, be ambitious to transpose them to the circumstances of the current pandemic. Nonetheless, both situations have a common feature, namely a public emergency threatening the life of the nation making it difficult to perform State functions in a normal way. In any event, we have seen that even in situations of distress for public authorities, in preserving the very essence of a right, the has Court required a valid explanation as to why a given interference with a human right has been necessary in the special circumstances of the case and what particular safeguards have been put in place.

## Conclusion

We saw how, in *A. and Others* and in *Kavala*, the emergency legislation at issue had been used by the respondent Governments in bad faith for purposes other than those initially claimed, even for the purposes of impeding the work of human rights defenders and cramping the application of the Convention. Cognisant of this danger, the Court adopted a strict interpretation of any restrictions imposed on human rights during public emergencies and clearly delimited the State Parties’ power, whereby not only the respect of non-derogable rights in Art. 15(2) of the Convention had to be ensured at all times, but also respect of all other Convention rights to a minimum extent. Art. 15 does not give State Parties a *carte blanche* to completely destroy derogable rights.^93^ Some minimum requirements have to be preserved at all times. Even without a derogation, the very essence of a right may not be extinguished in times of emergency and the Court’s case-law seems to indicate that basically all of the rights enshrined in the Convention are applicable almost in full during public emergencies.

In the present sanitary situation, the Court will have no difficulties in finding that there is an actual and imminent public emergency threatening the life of the nation. What will be difficult for Governments will be to prove whether the extraordinary measures taken during the COVID-19 period have been an adequate and proportionate response to the situation. Putting whole cities under lockdown in the first months of the pandemic may have been justified in the view of the unknown characteristics of the new virus and the lack of adequate preparedness and response. However, with the passage of time, when more evidence was surfacing as to how the virus spread and which protection measures were most effective, severe restrictions may no longer have been strictly required by the exigencies of the situation. Certainly, freedom of expression is one of those human rights which will be difficult to limit, unless it is used to spread misinformation. The pandemic does not allow Council of Europe Member States to take a relaxed attitude and to assume that any kind of restrictions on human rights will automatically be justified without a constant evaluation both of the situation and of the measures necessary to prevent further spread of the disease. It will be for the domestic courts to assess the situation on the ground and to ensure the protection of human rights in Council of Europe Member States in line with the Court’s case-law.

## Footnotes

1 Doswald-Beck, L.: Human rights in times of armed conflict and terrorism, Oxford University Press, Oxford [4], p. 69.

2 Harris, D.J, O’Boyle, M., Bates, E.P., Buckley, C.M.: Law of the European Convention on Human Rights, Second Edition, Oxford University Press [5], p. 620-621.

3 The text of the declarations is available at: https://www.coe.int/en/web/conventions/full-list/-/conventions/webContent/62111354.

4 Albanian Government declaration relating to the ECHR, 1 April 2020, available at: https://rm.coe.int/16809e0fe5.

5 Latvian Government declaration relating to the ECHR, 16 March 2020, available at: https://rm.coe.int/16809ce9f2.

6 North Macedonian Government declaration relating to the ECHR, 2 April 2020, available at: https://rm.coe.int/16809e1288.

7 Estonian Government declaration relating to the ECHR, 20 March 2020, available at: https://rm.coe.int/16809cfa87.

8 Georgian Government declaration relating to the ECHR, 23 March 2020, available at: https://rm.coe.int/16809cff20.

9 Moldovan Government declaration relating to the ECHR, 20 March 2020, available at: https://rm.coe.int/16809cf9a2.

10 Armenian Government declaration relating to the ECHR, 20 March 2020, available at: https://rm.coe.int/16809cf885.

11 Romanian Government declaration relating to the ECHR, 18 March 2020, p. 4, available at: https://rm.coe.int/16809cee30.

12 *Ibid*., p. 16-17.

13 Council of Europe, Preparatory work on Article 15 of the European Convention on Human Rights, Strasbourg, 27 January 1977, p. 4-6, available at: https://www.echr.coe.int/LibraryDocs/Travaux/ECHRTravaux-ART15-CDH(77)5-BIL1338902.pdf [3].

14 *Ibid*., p. 7.

15 *Ibid*., p. 15.

16 *Lawless v. Ireland* (No. 3), no. 332/57, 14 November 1960, § 28, A1.

17 *Ibid*.

18 From July 1954 to October 1999 the Convention judicial supervision system had a two-tier structure: the European Commission of Human Rights and the European Court of Human Rights.

19 *Denmark, Norway, Sweden and the Netherlands v. Greece*, nos. 3321/67, 3322/67, 3323/67 and 3344/67, Commission’s report of 5 November 1969, Yearbook 12, p. 70, § 113 (the Greek case).

20 *Ireland v. the United Kingdom*, no. 5310/71, 18 January 1978, § 207, A25.

21 *Ibid*.

22 *Ibid*., § 214.

23 *Ibid*., § 220.

24 *A. and Others v. the United Kingdom* [GC], no. 3455/05), 19 February 2009, § 174, ECHR 2009.

25 *Ibid*., § 177.

26 *Ibid*., § 178.

27 *Ibid*., § 179.

28 *Ibid*., § 180-181.

29 As of August 2020, more than 20 million cases with almost 1 million deaths worldwide. The most recent data is available on the World Health Organisation’s website: https://covid19.who.int/.

30 Secretary General of the Council of Europe, Respecting democracy, rule of law and human rights in the framework of the COVID-19 sanitary crisis, A toolkit for member states, SG/Inf(2020)11, 7 April 2020, available at: https://rm.coe.int/sg-inf-2020-11-respecting-democracy-rule-of-law-and-human-rights-in-th/16809e1f40 [6]; Committee of Ministers Declaration on the COVID-19 pandemic, 22 April 2020, available at: https://search.coe.int/cm/Pages/result_details.aspx?ObjectId=09000016809e33dd [1]; Commissioner for Human Rights Statement of 3 June 2020, available at: https://www.coe.int/en/web/commissioner/-/effectively-responding-to-a-sanitary-crisis-in-full-respect-for-human-rights-and-the-principles-of-democracy-and-the-rule-of-lawm [2].

31 The *Lawless* case [16], § 36.

32 *Ibid*., § 37.

33 *Brannigan and Mcbride v. the United Kingdom*, nos. 14553/89 and 14554/89, 26 May 1993, §§ 31, 33 and 48, A258-B.

34 *Ibid*., § 43.

35 *Ibid*., § 58.

36 *Ibid*., § 59.

37 *Ibid*., §§ 63-65.

38 *Aksoy v Turkey*, no. 21987/93, 18 December 1996, §§ 72 and 78, Reports of Judgments and Decisions 1996-VI.

39 *Ibid*., § 78.

40 *Ibid*., §§ 83-84.

41 *A. and Others v. the United Kingdom* [24], § 186-190.

42 *Ibid*., § 216.

43 *Ibid*., § 217.

44 *Ibid*., § 218.

45 *Ibid*., § 220.

46 *Mehmet Hasan Altan v. Turkey*, no. 13237/17, 20 March 2018, §§ 81 and 89-94.

47 *Ibid*., §§ 36, 129 and 140.

48 *Ibid*., § 139.

49 *Ibid*., § 149.

50 *Ibid*., §§ 164-165.

51 *Ibid*., § 166.

52 *Ibid*., § 207.

53 *Ibid*., §§ 188-193 and 209.

54 *Ibid*., §§ 209-210.

55 *Ibid*.

56 *Ibid*. § 211.

57 *Ibid*.

58 See, most recently, *Stomakhin v. Russia*, no. 52273/07, 9 May 2018, § 129; *Sallusti v. Italy*, no. 22350/13, 7 March 2019, § 62; *Stern Taulats and Roura Capellera v. Spain*, nos. 51168/15 and 51186/15, 13 March 2018, § 42.

59 *Zana v. Turkey* [GC], no. 18954/9, 25 November 1997, §§ 51 and 55-61, Reports 1997-VII.

60 *Alparslan Altan v. Turkey*, no. 12778/17, 16 April 2019, §§ 102 and 104-115.

61 *Ibid*., § 102.

62 *Ibid*., §§ 116-119.

63 *Ibid*., §§ 132-145.

64 *Ibid*., §§ 146-148.

65 *Baş v. Turkey*, no. 66448/17, 3 March 2020, §§ 183-184.

66 *Ibid*., §§ 188 and 200-201.

67 *Ibid*., § 221.

68 *Ibid*., § 224.

69 *Kavala v. Turkey*, no. 28749/18, 10 December 2019, §§ 15-16.

70 *Ibid*., § 28.

71 *Ibid*., §§ 139-146.

72 *Ibid*., § 157.

73 *Ibid*., §§ 217-232. The Court took account of, in particular, the way the prosecutor conducted the applicant’s interview and how he justified his indictment, largely, based on lawful acts; how the investigation was conducted, namely the timing when the applicant was arrested and statements of the head of State concerning his case.

74 Harris et al. [2], p. 632.

75 *Brogan and Others v. the United Kingdom*, nos. 11209/84, 11234/84, 11266/84, 11386/85, 29 November 1988, § 48, A145-B.

76 *Ibid*., § 59.

77 *Ibid*., § 62.

78 See p. 7.

79 *Sakik and Others v. Turkey*, nos. 23878-23883/94, 26 November 1997, Reports 1997-VII. The applicants were detained in an area which was not officially covered by the Turkish derogation. The Court, therefore, refused to apply the derogation in respect of facts which had happened in territory not covered by it.

80 *Ibid*., § 44.

81 *Ibid*., §§ 42-46.

82 *Zana v. Turkey* [59], §§ 51 and 55.

83 *Ibid*., §§ 52-62.

84 *Sürek v. Turkey*, no. 26682/95, 8 July 1999, § 61, Reports of Judgments and Decisions 1999-IV.

85 *Ibid*.

86 *Ibid*., §§ 62-65.

87 *Al-Skeini and Others v. the United Kingdom* [GC], no. 55721/07, 7 July 2011, § 161, Reports of Judgments and Decisions 2011.

88 *Ibid*., §§ 164-177.

89 *Jaloud v. The Netherlands* [GC], no. 47708/08, 20 November 2014, §§ 226-228, Reports of Judgments and Decisions 2014. To be noted that several aspects the Court’s assessment of the effectiveness of the investigation were heavily criticised by seven judges. See Joint concurring opinion of judges Casadevall, Berro-Lefevre, Šikuta, Hirvelä, López Guerra, Sajó and Silvis.

90 *Khlebik v. Ukraine*, no. 2945/16, 25 July 2017, §§ 65 and 81.

91 *Ibid*., §§ 71 and 74.

92 *Ibid*., §§ 73-81.

93 Doswald-Beck [3] p. 70.
